# Synergistic control of viral persistence via wettability and ion release on antiviral coatings

**DOI:** 10.1016/j.mtbio.2026.103449

**Published:** 2026-07-13

**Authors:** Ryohei Hirose, Saori Morita, Shizuka Kanawa, Akinobu Sai, Taku Kano, Takumi Minamiyama, Satomi Isono, Takaaki Nakaya

**Affiliations:** a Department of Infectious Diseases, Graduate School of Medical Science, Kyoto Prefectural University of Medicine, Japan; b Department of Molecular Gastroenterology and Hepatology, Graduate School of Medical Science, Kyoto Prefectural University of Medicine, Japan; c Tokyo Research Center, HardoLass Holdings Co., Ltd., Japan; d Department of Forensic Medicine, Graduate School of Medical Science, Kyoto Prefectural University of Medicine, Japan

**Keywords:** Antiviral coatings, Surface wettability, Viral persistence, Metal ion release, Biointerfaces

## Abstract

Antiviral coating design remains largely empirical due to the lack of quantitative principles linking interfacial properties to viral persistence under realistic conditions. Here, we establish a survival time–based evaluation framework that captures the drying dynamics of 2-μL virus-containing microdroplets and enables quantitative assessment of antiviral coating performance using viral survival time (duration of infectivity) and survival time reduction rate.

Using this framework, we identify surface virus density, defined as infectious virus per unit distribution area, as a physical determinant governing viral persistence. When surface virus density was matched, viral survival times converged irrespective of inoculum level or droplet geometry. Accordingly, viral survival time decreased with decreasing surface virus density, and hydrophilic surface design alone reduced viral survival time by approximately 70–80%.

Furthermore, we demonstrate that the initial release rate of antiviral metal ions constitutes a complementary chemical control parameter under rapid-drying conditions. Antiviral coatings incorporating amorphous vanadate glass particles enabled rapid release of copper or silver ions within the first 1–5 min following droplet deposition. By integrating wettability-mediated viral redistribution with rapid ion release, synergistic suppression of viral persistence was achieved, reducing viral survival time relative to uncoated surfaces by up to 97.6% for influenza virus, 98.2% for feline calicivirus, and 94.9% for highly pathogenic avian influenza virus.

These findings demonstrate that viral persistence can be predictively controlled through the combined physical regulation of surface virus density and chemical inactivation by rapidly released antiviral agents. This antiviral coating strategy may contribute to reducing contact transmission risks in healthcare, agricultural, and community environments.

## Highlights

• Viral survival time enables endpoint-independent performance evaluation.
• Surface virus density is identified as a governing variable of viral persistence.
• Hydrophilic surface design alone reduces viral survival time by 70–80%.
• Early-stage ion release governs antiviral efficacy under drying conditions.
• Coupled wettability and ion release reduce viral persistence by up to 98%.

## Graphical abstract

**Figure:**
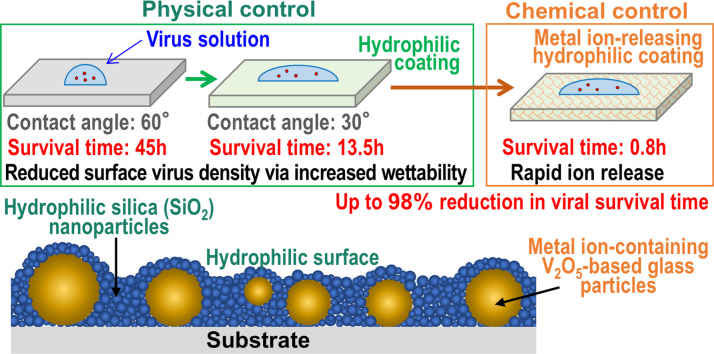

## 1 Introduction

Understanding how infectious viruses persist at material interfaces is essential for mitigating contact transmission in healthcare and community environments; however, a unifying quantitative framework that explains how interfacial properties govern viral persistence remains lacking [[1], [2], [3]]. Because the risk of contact transmission scales with the amount of infectious virus remaining on surfaces [[4], [5], [6]], identifying the determinants governing viral persistence is critical. Numerous studies have investigated the temporal changes in viral persistence on various environmental surfaces, including plastics, glass, and metals [[7], [8], [9], [10]]. In recent years, efforts have further expanded toward the development of antiviral surfaces that actively reduce the amount of residual virus [[11], [12], [13], [14]]. Such antiviral surfaces have been realized not only through material design, such as alloys and polymeric materials, but also through surface modification approaches, including coatings, which have been extensively studied and implemented in practical applications [[13], [14], [15], [16]]. Among these, coating-based and nanostructured antiviral surfaces have attracted considerable attention because they enable tunable control of interfacial physicochemical properties and pathogen–surface interactions through rational materials design [[17], [18], [19], [20], [21], [22], [23], [24], [25]]. Under appropriately designed conditions, antiviral functionality can be imparted to a wide range of substrates regardless of the base material, establishing antiviral coatings as a versatile and practical strategy across diverse functional interfaces [18,26,27].

**Fig. 1:**
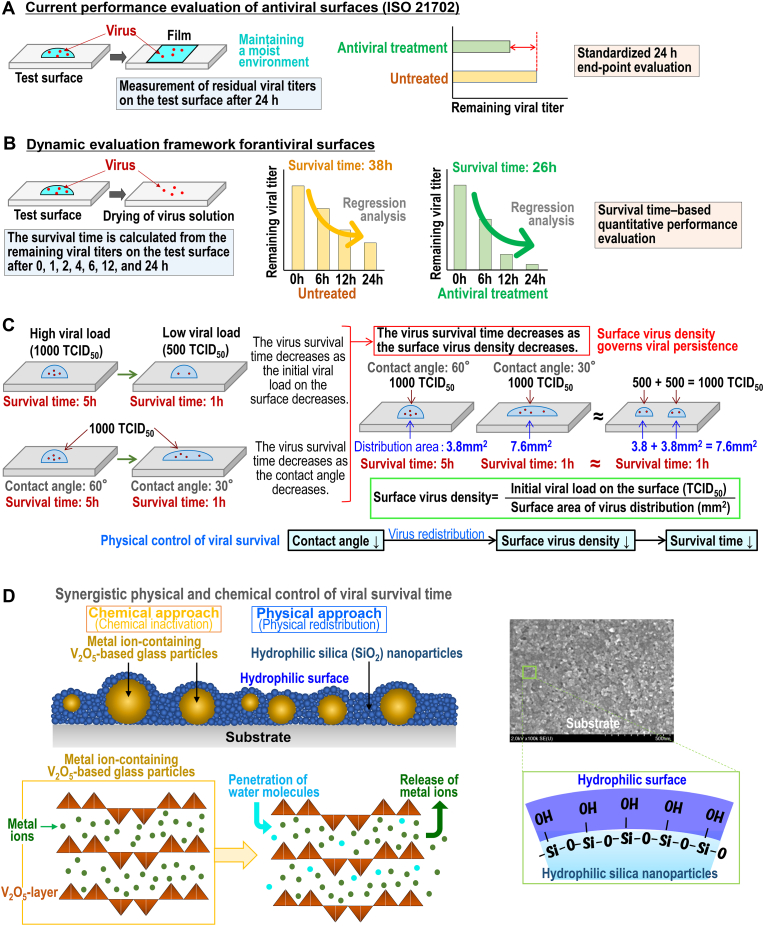
**(A) Current standardized evaluation of antiviral surfaces (ISO 21702) under persistently wet conditions.** The assay is designed for standardized comparison of antiviral performance under controlled wet conditions; however, drying dynamics and air–liquid interface formation are not included in the evaluation framework. In addition, antiviral efficacy is assessed as virus reduction at a single 24 h end point. **(B) Proposed performance evaluation framework for antiviral surfaces.** This approach simulates real-world environments more closely and quantifies antiviral efficacy using dynamic parameters, namely survival time and the survival time reduction rate. This enables accurate and objective evaluation and comparison of antiviral surface performance. **(C) Surface virus density as a governing determinant of viral survival time.** When viral load is normalized by distribution area, survival times converge across different inoculum volumes and contact angles, revealing a unifying physical principle governing viral persistence on surfaces. **(D) Synergistic physical and chemical control of viral survival on antiviral coating surfaces.** Materials design strategy combining wettability-mediated viral redistribution with rapid metal-ion release.

Despite these advances, important gaps remain in establishing quantitative and predictive design principles for antiviral surfaces. First, the current standard for evaluating antiviral surface performance (ISO 21702), while highly valuable for standardized performance assessment, was not specifically designed to reproduce all aspects of infection-relevant environmental contamination scenarios. ISO 21702 provides an important standardized method for comparing antiviral surface performance under controlled experimental conditions. Because the method employs persistently wet conditions and a fixed endpoint, its primary purpose is standardized benchmarking rather than quantification of viral persistence dynamics under drying environments [[26], [27], [28], [29], [30], [31]]. Furthermore, reliance on a single 24 h endpoint limits the kinetic information available for analyzing viral inactivation dynamics. Consequently, additional evaluation approaches may be useful for quantitatively linking interfacial properties to viral persistence and for supporting the rational design of antiviral coatings under realistic drying conditions (Fig. 1A).

Second, the mechanistic variables governing antiviral surface performance remain insufficiently defined and lack quantitative integration. For antiviral coatings that rely on release of active agents, a systematic understanding of the optimal antiviral agent and the corresponding release kinetics remains limited. Studies have demonstrated that metal ion release behavior critically influences antiviral efficacy; however, comprehensive quantitative relationships linking release kinetics to antiviral performance remain lacking [13,[32], [33], [34]]. Additionally, even in the absence of antiviral agents, clear design rules describing which surface properties suppress viral survival remain lacking. We have previously reported that physical surface properties, such as water contact angle, may influence viral survival; however, the underlying mechanisms remain unclear [[35], [36], [37], [38], [39]]. Importantly, although previous studies reported correlations between wettability and viral persistence, no study has identified and experimentally validated a quantitative governing variable that unifies these observations across different droplet geometries and inoculum conditions. Collectively, these limitations stem from the lack of evaluation methods capable of dynamically and quantitatively assessing antiviral performance, thereby hindering the development of predictive interface engineering strategies for antiviral coatings.

In this study, we first establish a quantitative evaluation framework that captures realistic microdroplet drying conditions, namely, virus-containing microdroplets that rapidly dry on surfaces under practical environmental conditions (Fig. 1B). To this end, we adapt a previously developed model that quantifies viral survival time on surfaces, defined as the duration over which viruses retain infectivity. The survival time reduction rate is introduced as a quantitative metric for comparing antiviral coating performance.

Next, from an interface engineering perspective, we systematically elucidate how surface properties govern viral survival time. Previous studies have reported correlations between surface properties, particularly contact angle, and viral survival time. However, the quantitative variable underlying these relationships has remained unclear. Building on this, we test the hypothesis that surface virus density—defined as infectious viral load per unit distribution area—serves as a key physical determinant and unifying quantitative variable governing viral survival time (Fig. 1C). Furthermore, leveraging these insights, we construct a matrix-driven antiviral coating that exhibits antiviral functionality solely through modulation of surface wettability, without the use of antiviral agents.

In addition, for metal ion-releasing antiviral coatings, we quantitatively investigate how release kinetics and cumulative ion release govern reductions in viral survival time. Various classes of antiviral agents have been incorporated into antiviral coatings, including metal ions, metal nanoparticles, and organic compounds such as quaternary ammonium salts and amine-modified silanes [34,40,41]. However, organic antiviral agents often show limited efficacy against non-enveloped viruses and reduced durability [40,42]. By contrast, metal ions such as copper and silver are effective against both enveloped and non-enveloped viruses, offering broad-spectrum antiviral activity along with superior environmental stability and rapid action [32,43,44]. In this study, we focus on metal ion–releasing coatings as a representative release-based antiviral strategy and establish quantitative relationships between ion release kinetics and antiviral performance under drying conditions.

Ultimately, this study establishes surface virus density as a governing determinant of viral persistence on solid surfaces and defines quantitative design rules for antiviral coating engineering. By integrating physical control through surface virus density with chemical control through antiviral agent release, we demonstrate a high-performance antiviral coating strategy capable of predictably suppressing viral persistence through the synergistic combination of physical redistribution and chemical inactivation. These findings provide quantitative design principles for the development of next-generation antiviral coatings and functional interfaces under realistic environmental conditions.

## 2 Materials and methods

### 2.1 Preparation of viruses and cells

To enable standardized comparison with established benchmarks, feline calicivirus (FCV) as a non-enveloped virus and human influenza virus (IFV) as an enveloped virus—both defined in ISO 21702—were employed for the antiviral performance evaluation.

Madin-Darby canine kidney (MDCK) and Crandell Reese feline kidney (CRFK) cells were purchased from the Riken BioResource Center Cell Bank (Ibaraki, Japan), and cultured in minimal essential medium (MEM) (Sigma Aldrich, St Louis, MO, USA), supplemented with 10% fetal bovine serum (FBS) and standard antibiotics (penicillin/streptomycin/amphotericin B) [7,45].

Influenza A virus (IFV; A/Puerto Rico/8/1934 [H1N1], VR-1469) and feline calicivirus (FCV; VR-782) were obtained from the American Type Culture Collection (ATCC, Manassas, VA, USA). IFV and FCV stocks were propagated in 9-day-old embryonated chicken eggs and CRFK cells, respectively. Each virus was then concentrated and purified as follows: the culture medium was centrifuged at 2500 ×g for 10 min at 4 °C to remove debris, and the supernatant was sterilized by filtration using a 0.45 μm filter. Each virus sample in the supernatant was then ultracentrifuged in a Beckman SW28 rotor (Beckman Coulter Inc, Brea, CA) at 26000 rpm for 2.5 h at 4 °C and pelleted through a 20% (w/w) sucrose cushion. Subsequently, each virus was stored frozen at −80 °C until further use. Each viral suspension was diluted to the required concentration in phosphate-buffered saline (PBS; Nacalai Tesque) [7,45].

IFV and FCV titers were determined as the 50% tissue culture infectious dose (TCID_50_) in MDCK and CRFK cells, respectively. Specifically, MDCK or CRFK cells cultured in 96-well plates were inoculated with IFV or FCV, and after 3–4 days of inoculation, the cytopathic effect (CPE) in each well was assessed using an inverted optical microscope (Olympus IX71; Olympus, Tokyo, Japan), and TCID_50_/ml was calculated using the Behrens–Karber method [45,46]. The detection limit for IFV and FCV titers was 10°^.5^ TCID_50_/ml.

In this study, avian influenza viruses (AIVs) were also included as targets for evaluating the performance of the antiviral surfaces. Highly pathogenic avian influenza virus AIV-H5N1 (A/crow/Kyoto/53/04; H5N1) and low pathogenic avian influenza virus AIV-H5N3 (A/Duck/Hong Kong/820/80; H5N3) were propagated in 9-day-old embryonic chicken eggs [47,48]. The allantoic fluids were precleared by centrifugation at 3300 × g for 30 min and subsequent filtration through a 0.45 μm filter to remove impurities in advance. Finally, virus in the allantoic fluid was purified through a 20% (w/v) sucrose cushion in PBS by ultracentrifugation at 26,000 rpm for 2.5 h at 4 °C in a Beckman SW28 rotor [47]. The virus pellets were suspended in PBS and stored at −80 °C as a working stock. AIV-H5N1 and AIV-H5N3 titers were measured in terms of TCID_50_ in MDCK. Specifically, MDCK cells cultured in 96-well plates were inoculated with AIV, and after 3–4 days of inoculation, the CPE in each well was assessed using an inverted optical microscope (Olympus IX71), and TCID_50_/ml was calculated using the Behrens–Karber method. The detection limit for AIV titers was 10°^.5^ TCID_50_/ml. Experiments involving AIVs were conducted at Kyoto Prefectural University of Medicine under biosafety level 3+ conditions (approved by the Ministry of Agriculture, Forestry and Fisheries of Japan).

### 2.2 Preparation of coating surfaces with controlled contact angle

After mixing 10 mL of absolute ethanol (FUJIFILM Wako Pure Chemical Corporation), 5.6 mL of distilled water, and 0.4 mL of 1 N HCl (FUJIFILM Wako Pure Chemical Corporation), 4 g of ethyl silicate 40, a pentamer of triethoxysilane (Colcoat Company Limited, Tokyo, Japan), was added. The mixture was stirred at room temperature for at least 2 h to obtain a hydrolyzed triethoxysilane solution. This solution was then added to an inorganic coating solution (EXCEL PURE BD-P05; Central Automotive Products Ltd, Osaka, Japan) to final concentrations of 1.5, 2, 3, 5, and 10 wt%, followed by stirring at room temperature. This procedure yielded coating solutions of varying hydrophilicity, enabling the preparation of surfaces with different water contact angles [49].

Subsequently, 2 mL of each coating solution was dropped onto the surface of 5 × 5 cm glass substrates and spin-coated at 1000 rpm for 20 s using a spin coater (MS-B300; Mikasa Company Limited, Tokyo, Japan). The coated glass substrates were then dried at room temperature for 1 h. Consequently, coating surfaces with water contact angles of 20, 30, 40, 50, and 60°, measured 30 s after droplet deposition, were obtained.

### 2.3 Preparation of coating surfaces that release metal ions

Vanadate glass particles (0.4 g) were added to 39.6 g of an inorganic coating solution (EXCEL PURE BD-P05), and the mixture was homogenized for 2 min using a planetary centrifugal mixer (AR-100; Thinky Corporation, Tokyo, Japan). This procedure yielded an inorganic coating solution containing vanadate glass particles [50].

Subsequently, approximately 2 mL of the prepared coating solution was dropped onto the surface of 5 × 5 cm glass substrates and spin-coated at 500 rpm for 10 s using a spin coater (MS-B300). The coated glass substrates were then dried at room temperature for 1 h.

All coated surfaces were adjusted to exhibit a water contact angle of 30°, measured 30 s after droplet deposition. The type, release rate, and total amount of metal ions released from the coatings were controlled by the composition of the vanadate glass particles incorporated into the inorganic coating solution. Specifically, vanadate glass particles with a composition of V_2_O_5_–P_2_O_5_–BaO–CuO were used to prepare coatings with a high copper ion release, whereas particles with a composition of V_2_O_5_–P_2_O_5_–CuO–Fe_2_O_3_ were used to prepare coatings with a low copper ion release. Similarly, vanadate glass particles with a composition of V_2_O_5_–P_2_O_5_–BaO–Ag_2_O were used to prepare coatings with a high silver ion release, while particles composed of V_2_O_5_–P_2_O_5_–Ag_2_O–Fe_2_O_3_ were used to prepare coatings with a low silver ion release. The rapid release behavior is attributed to the amorphous structure of the vanadate glass particles, which facilitates ion diffusion within the hydrophilic coating matrix (Fig. 1D).

### 2.4 Contact angle measurement

Based on the wettability test JIS R3257 [51], the contact angle of distilled water on each coated surface was measured using a contact-angle meter (LSE-ME5, NIC Corporation, Saitama, Japan). Specifically, in an indoor environment (temperature 25 ± 5 °C, humidity 50 ± 10%), 2 μL of distilled water was placed on each coated surface, and the contact angle was monitored for 30 s. The contact angle was measured five times independently at different locations on the same surface. The contact angle measured 30 s after droplet deposition was used for subsequent analyses.

### 2.5 Viral survival assessment on each coated surface (antiviral performance evaluation)

Viral survival time (i.e., the duration over which viruses retain infectivity) was evaluated for each coated surface (Fig. 1B) [7,46].

Specifically, IFV or FCV was suspended in PBS and applied in 2-μL aliquots to each surface (amount of virus: 1.0 × 10^5^ TCID_50_). Uncoated glass surfaces were used as control surfaces throughout the study. The surfaces were then placed in a constant temperature and humidity chamber, and incubated under controlled conditions (25 °C, 50% relative humidity) for durations ranging from 0, 5, 10, 30, 60, 120, 240, 360, 720, 1080, 1440, and 2880 min. Complete visual dryness of the 2-μL aliquot was observed within a 5–10 min window. After incubation, the residual virus on the surface was recovered in 1 mL of MEM with 10% FBS and 1 mM ethylenediaminetetraacetic acid (EDTA), and titrated. EDTA was added to chelate metal ions eluted from the coated surface. For each measurement, three independent experiments were performed, and the results were expressed as the mean ± standard error of the mean. These experimental conditions were selected to mimic small respiratory droplets deposited onto environmental surfaces under typical indoor conditions. A droplet volume of 2 μL was adopted as a representative microdroplet condition because small contaminating droplets are more likely to remain unnoticed on environmental surfaces and may therefore contribute to contact transmission in the absence of cleaning or disinfection interventions.

Zero-hour incubation refers to virus recovery immediately after application of the virus mixture to each surface. For quality control, the recovered viral titer under the zero-hour incubation condition was confirmed to be within 5.0 ± 0.25 log_10_ TCID_50_. Experiments falling outside this predefined range were repeated. In addition, the duration of observation was selected so that infectious virus titers declined below the detection limit under all tested conditions, thereby minimizing uncertainties associated with extrapolation of survival time. As a supplementary note, viral survival assessments on coated surfaces with water contact angles adjusted to 20–60° were exceptionally conducted under conditions of 20 °C and 65% relative humidity, as these conditions prolonged viral persistence and improved discrimination of survival time differences among surfaces with varying wettability.

Data were analyzed using GraphPad Prism 7 (GraphPad, Inc., La Jolla, CA, USA). Log-transformed elapsed time was used as the explanatory variable (X-axis), and log-transformed virus titer as the response variable (Y-axis). A linear regression analysis with a logarithmic link function was performed for each virus to generate a regression model. The detection limit of the IFV and FCV titers was 10°^.5^ TCID_50_. Therefore, estimated survival time was obtained from the fitted regression model as the time required for viral infectivity to decline to the detection limit, corresponding to the X value at Y = 0.5 on the regression curve. Estimated survival times are presented with 95% confidence intervals derived from the regression models. The half-lives of IFV and FCV were estimated from the slope of each regression curve when the titers of viruses remaining on the surface were 2.0 and 3.0 log_10_ TCID_50_, respectively. The survival time reduction rate, an indicator of antiviral surface performance, was calculated using the following formula.

$$Survival\;time\;reduction\;rate=100\times \frac{Survival\;time\;on\;control\;surface-survival\;time\;on\;coated\;surface}{Survival\;time\;on\;control\;surface}$$

### 2.6 Measurement of copper and silver ion elution from coating surfaces and kinetic modeling

#### 2.6.1 Preparation of calibration curves

Copper and silver ion calibration curves were prepared using standard solutions. Copper ion standard solutions (0.1, 1, 3, and 5 mg/L) were prepared from copper (II) sulfate pentahydrate (CuSO_4_⋅5H_2_O; FUJIFILM Wako Pure Chemical Corporation, Osaka, Japan) diluted with deionized water and quantified using PONAL KIT-Cu (Dojindo Laboratories, Kumamoto, Japan). Absorbance was measured at 482 nm using a UV–Vis–NIR spectrophotometer (UH5700; Hitachi High-Tech Corporation, Tokyo, Japan).

Silver ion standard solutions (0.1, 1, 3, and 5 mg/L) were prepared by diluting a commercial silver standard solution (1000 mg/L; FUJIFILM Wako Pure Chemical Corporation) with ultrapure water. After color development using phosphate, manganese sulfate, and potassium peroxodisulfate, absorbance was measured at 525 nm. Calibration curves were constructed by plotting absorbance against ion concentration for both copper and silver ions.

#### 2.6.2 Elution experiments and quantification

To evaluate ion release from coating surfaces, coating samples were immersed in deionized water for predetermined durations (1, 3, 5, and 10 min, and 6, 15, and 24 h). For copper ion measurements, 5 × 5 cm samples were immersed in 5 mL of deionized water. For silver ion measurements, 4 × 5 cm samples were immersed in 4 mL of deionized water. At each time point, the coating samples were removed and the concentrations of released ions were quantified using the corresponding calibration curves.

The cumulative ion elution per unit area, *Q(t)* (μg/cm^2^), was calculated using Equation (1):

(1) $$Q\left(t\right)=C\left(t\right)\times \frac{V}{A}\times 1000$$

where *C(t)* is the ion concentration (mg/L), *V* is the liquid volume (mL), and *A* is the coating surface area (cm^2^). This value was defined as the ion elution capacity of the coating surface.

#### 2.6.3 Kinetic modeling and estimation of droplet-scale concentrations

To quantitatively characterize ion release behavior, a kinetic analysis was performed using a first-order saturation model. Preliminary experiments demonstrated that both copper and silver ion release reached a plateau between 360 and 900 min. Therefore, the ion concentration measured at 900 min was defined as the saturation concentration (*C*_max_) and used for kinetic modeling.

The early-stage release data (1, 3, 5, and 10 min) were fitted using the exponential saturation model shown in Equation (2):

(2) $$C\left(t\right)=C_{\max}\left(1-e^{-\frac{k\cdot t\cdot A}{V}}\right)$$

where *k* represents the apparent ion release rate constant.

To estimate ion concentrations under antiviral testing conditions, ion release into a microdroplet environment (*V*_*drop*_ = 2 μL, *A*_*contact*_ = 7.5 mm^2^) was calculated by substituting the corresponding surface-area-to-volume ratio into Equation (2). Concentrations at 1, 3, and 5 min were estimated for both copper and silver ions. When extrapolated values exceeded *C*_max_ because of the extremely high surface-area-to-volume ratio, the calculated concentration was capped at *C*_max_ to reflect physical equilibrium.

Because the virus suspension used in this study was prepared by diluting virus stocks more than 1000-fold in PBS and contained no additional organic components, ion release behavior was assumed to approximate that observed in deionized water.

### 2.7 Evaluation of antiviral activities of aqueous copper ion or silver ion solutions at various concentrations

Copper (II) chloride (Nacalai Tesque) was dissolved in distilled water to prepare aqueous solutions with copper ion concentrations of 10, 100, and 1000 μg/dL. Silver nitrate (Nacalai Tesque) was similarly dissolved in distilled water to prepare aqueous solutions with silver ion concentrations of 10, 100, and 1000 μg/dL. In a 1.5-mL tube, 10 μL of distilled water containing either IFV or FCV (1.0 × 10^5^ TCID_50_) was mixed with 90 μL of the aqueous copper or silver ion solutions for 1, 3, 5, 10, and 30 min. Subsequently, the resulting solutions were neutralized with 900 μL of MEM with 10% FBS and 1 mM EDTA, and the remaining viral titers were measured [36,52]. The detection limit for both IFV and FCV was 10°^.5^ TCID_50_. To evaluate the antiviral activity under each condition, we calculated the log reductions of the viral titers, with normalization to the distilled water control. Three independent experiments were performed for each condition, and the results were expressed as the mean ± standard error of the mean.

### 2.8 Statistical analysis

Data obtained were analyzed using GraphPad Prism 7 (GraphPad Inc.). To analyze the relationship between virus density and survival time, we used Pearson's correlation coefficient between the virus density and logarithm of survival time. Continuous variables were evaluated using Student's t-test. All reported p-values were two-sided, and values with p < 0.05 were considered significant.

## 3 Results

### 3.1 Relationship between surface virus density and viral survival

**Fig. 2:**
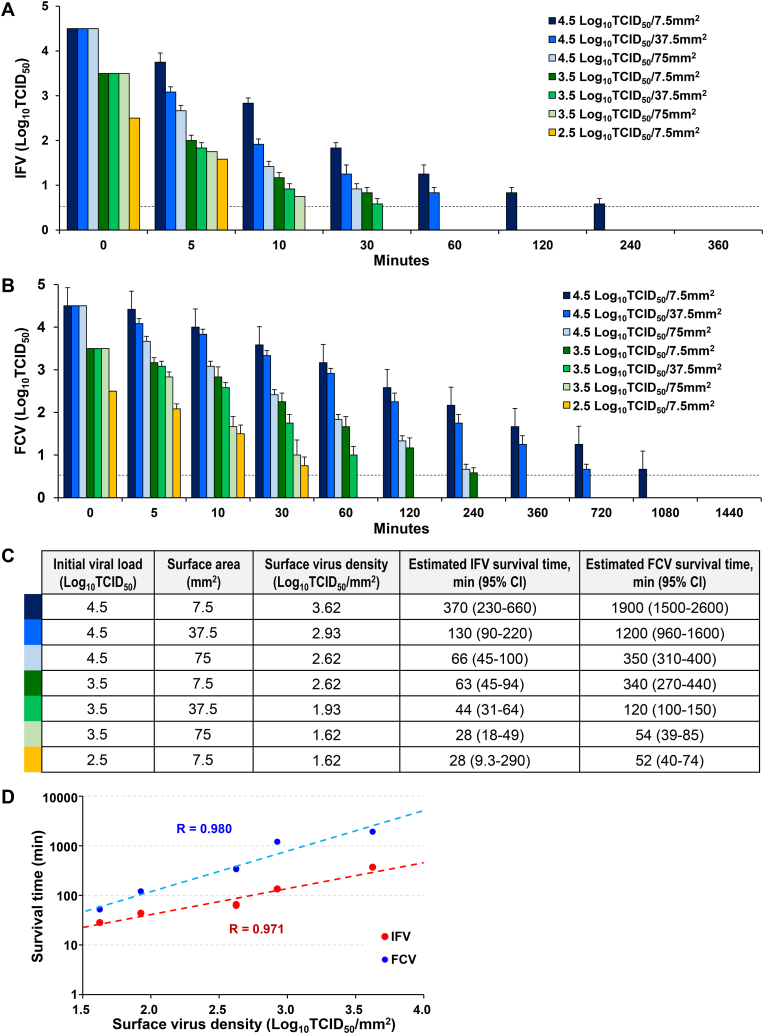
**Viral titer kinetics and their correlation with surface virus density. (A, B)** Time-course changes in the titers of **(A)** influenza virus (IFV) and **(B)** feline calicivirus (FCV) remaining on surfaces under various inoculation conditions. Viral titers were measured over time on uncoated glass surfaces by varying the initial viral load and the inoculation surface area. For each measurement, three independent experiments were conducted, and the results are expressed as the mean ± standard error. Data points below the detection limit are omitted, and dotted horizontal lines indicate the detection limit titers. **(C) Surface virus density and survival times of IFV and FCV under all seven inoculation conditions. (D) Correlation analysis between surface virus density and viral survival time.**

As the initial viral inoculum applied to glass surfaces decreased from 4.5 to 3.5 and 2.5 log_10_ TCID_50_, the amounts of infectious IFV and FCV remaining on the surface at each time point decreased accordingly. In addition, under conditions with the same initial viral inoculum, increasing the inoculated surface area from 7.5 to 37.5 and 75 mm^2^ resulted in lower amounts of infectious IFV and FCV remaining on the surface at each time point (Fig. 2A and B).

Based on the data shown in Fig. 2A and B, viral survival time and surface virus density were calculated (Fig. 2C and Supplementary Figs. S1 and S2). In this study, surface virus density was defined as the amount of infectious virus per unit surface area (TCID_50_/mm^2^), which is conceptually distinct from the conventional definition of viral load (Fig. 1C). As the initial viral inoculum decreased from 4.5 to 3.5 and 2.5 log_10_ TCID_50_, the survival times of both IFV and FCV were shortened. Similarly, increasing the inoculated surface area from 7.5 to 37.5 and 75 mm^2^ resulted in shorter survival times for both viruses. Specifically, under conditions with surface areas of 7.5 and 75 mm^2^, the survival times of IFV were 370 and 66 min, respectively, while those of FCV were 1900 and 350 min, respectively. The survival times of both IFV and FCV on surfaces with an area of 75 mm^2^ were significantly shorter than those on surfaces with an area of 7.5 mm^2^ (Fig. 2C). For both IFV and FCV, survival time decreased as surface virus density decreased. Importantly, when surface virus density was identical, survival times of IFV and FCV were nearly the same, even when the initial viral inoculum and inoculated surface area differed, and no significant differences were observed. For example, the condition with an initial viral inoculum of 4.5 log_10_ TCID_50_ applied to 75 mm^2^ and the condition with 3.5 log_10_ TCID_50_ applied to 7.5 mm^2^ yielded the same surface virus density of 2.62 log_10_ TCID_50_/mm^2^. Under these two conditions, the survival times of IFV and FCV were nearly identical, and no significant differences were observed. Similarly, no significant differences in survival time were observed between different conditions that shared the same surface virus density of 1.62 log_10_ TCID_50_/mm^2^ (Fig. 2C). These results indicate that viral survival on surfaces is governed not simply by the initial viral inoculum, but by the amount of virus distributed per unit surface area.

Finally, an extremely strong correlation was observed between the surface virus density and the logarithm of survival time, with correlation coefficients of 0.971 for IFV and 0.980 for FCV (both P < 0.01) (Fig. 2D).

### 3.2 Influence of surface wettability, defined by contact angle, on surface virus density and survival time

**Fig. 3:**
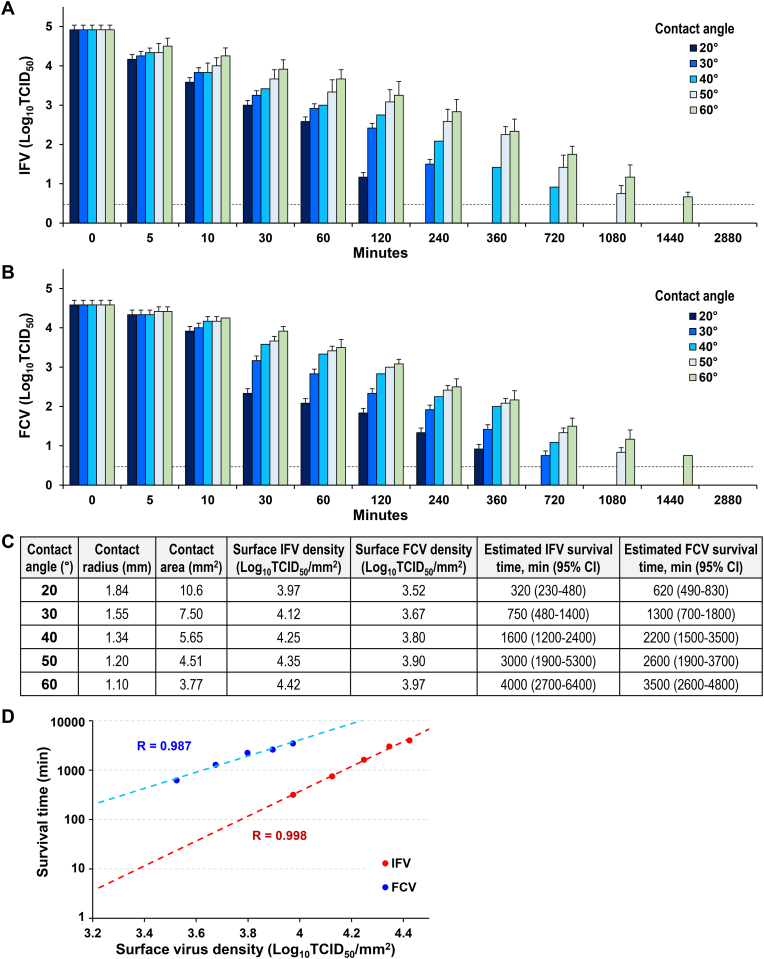
**Viral titer kinetics and their correlation with surface wettability (contact angle). (A, B)** Time-course changes in the titers of **(A)** influenza virus (IFV) and **(B)** feline calicivirus (FCV) remaining on coated surfaces with contact angles adjusted to 20, 30, 40, 50, and 60°. For each measurement, three independent experiments were conducted, and the results are expressed as the mean ± standard error. Data points below the detection limit are omitted, and dotted horizontal lines indicate the detection limit titers. **(C) Contact angle, contact radius, contact area, surface virus density, and viral survival times for all five coated surfaces. (D) Correlation analysis between surface virus density and viral survival time on the five coated surfaces.**

Viral survival was evaluated on coating surfaces with water contact angles adjusted from 20° to 60°. As the contact angle decreased, the amounts of infectious IFV and FCV remaining on the surfaces at each time point decreased accordingly (Fig. 3A and B). Viral survival times were calculated from the data shown in Fig. 3A and B, revealing that the survival times of both IFV and FCV were progressively shortened on surfaces with lower contact angles (Fig. 3C and Supplementary Figs. S3 and S4). Specifically, on surfaces with contact angles of 60° and 20°, the survival times of IFV were 4000 and 320, respectively, while those of FCV were 3500 and 620, respectively. Compared with surfaces with a contact angle of 60°, viral survival times on surfaces with a contact angle of 20° were significantly shorter for both IFV and FCV.

Because the inoculated virus droplet volume was intentionally fixed at 2 μL, lower contact angle surfaces resulted in a larger wetted surface area, leading to a reduced surface virus density (Fig. 3C). For both IFV and FCV, survival time decreased as surface virus density decreased, corresponding to decreasing contact angles. Furthermore, an extremely strong correlation was observed between the surface virus density and the logarithm of survival time, with correlation coefficients of 0.998 for IFV and 0.987 for FCV (both *P* < 0.01) (Fig. 3D).

Taken together, these results strongly support the hypothesis presented in Fig. 1C, demonstrating that surface virus density is a critical determinant of viral survival on surfaces.

### 3.3 Antiviral activity of copper and silver ions and their release behavior from coating surfaces

**Fig. 4:**
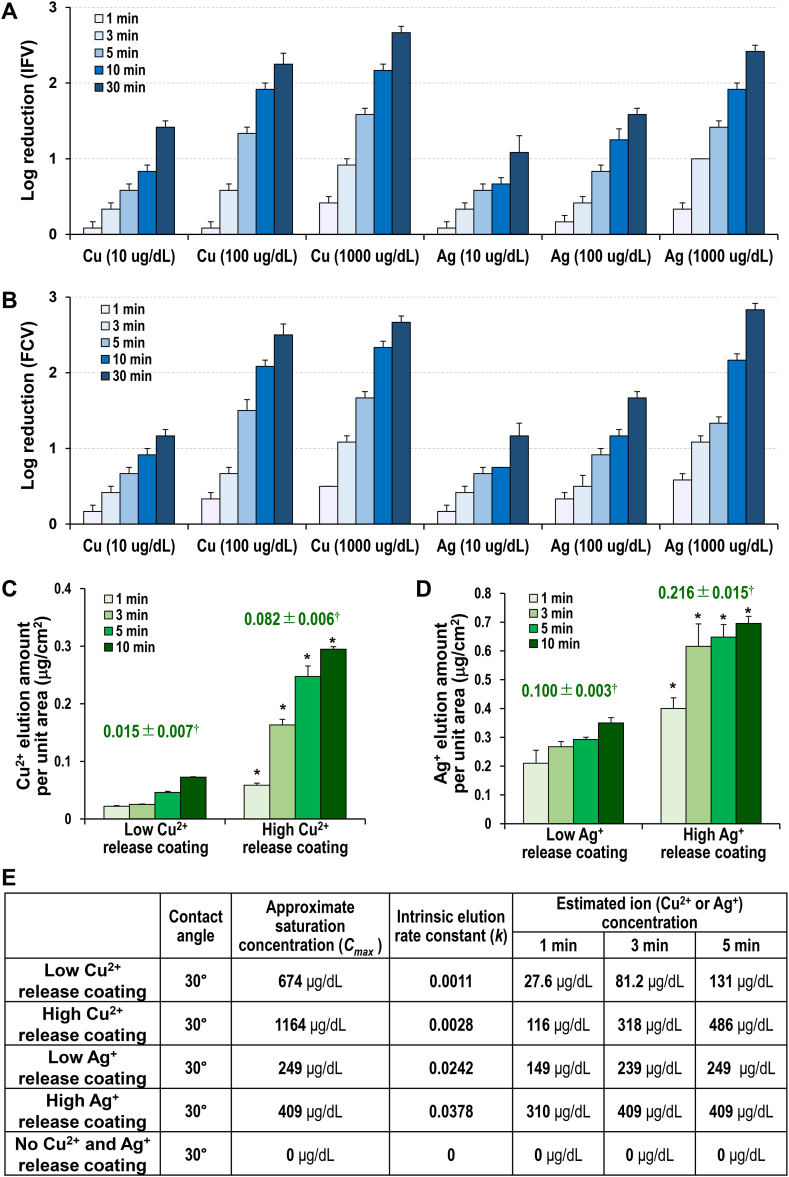
**(A, B) Antiviral activity of aqueous copper and silver ion solutions at various concentrations against (A) influenza virus (IFV) and (B) feline calicivirus (FCV).** Antiviral activity, expressed as log reduction values, was evaluated for aqueous solutions containing copper or silver ions at concentrations of 10, 100, and 1000 μg/dL. Log reductions in viral titers were calculated relative to the phosphate-buffered saline control. Three independent experiments were conducted for each condition, and the results are expressed as the mean ± standard error of the mean. **(C, D) Metal ion elution amount per unit area from antiviral coating surfaces.** Four types of antiviral coating surfaces were prepared: **(C)** two coatings with different copper ion release rates and **(D)** two coatings with different silver ion release rates. The amount of copper or silver ions eluted per unit area from each coating surface was measured. Based on these data, the elution rate of copper or silver ions during the initial elution phase (up to 5 min) was calculated. **^†^**Initial elution rate (μg/cm^2^·min). **∗***P* < 0.05 versus the corresponding low-release coating. **(E) For the four types of antiviral coating surfaces, contact angle, approximate saturation concentration of eluted metal ions (*C*_*max*_*)*, intrinsic elution rate constant (*k*), and estimated metal ion concentrations in a 2 μL droplet at each elapsed time.**

First, the antiviral activities of copper and silver ion solutions at 10, 100, and 1000 μg/dL were evaluated. For both IFV and FCV, antiviral activity, defined as the log reduction value, increased with increasing ion concentration and with longer reaction times. In addition, under a representative condition reflecting practical use—namely, a 5-min reaction with 100 μg/dL of copper or silver ions—copper ions exhibited significantly higher log reduction values than silver ions for both viruses (Fig. 4A and B).

In this study, four types of antiviral hydrophilic coating surfaces were prepared: two coatings with different copper ion release rates and two coatings with different silver ion release rates (Fig. 1D). The high-release coatings were designed to achieve the maximum ion release levels considered acceptable from the perspectives of product safety and durability for practical applications. During the initial elution phase (up to 5 min), the initial elution rates of Cu^2+^ were 0.015 ± 0.007 and 0.082 ± 0.006 μg/cm^2^·min for the low and high Cu^2+^ release coatings, respectively, indicating that copper ion release from the high-release coating was approximately sevenfold faster than that from the low-release coating (Fig. 4C). Similarly, the initial elution rates of Ag^+^ were 0.100 ± 0.003 and 0.216 ± 0.015 μg/cm^2^·min for the low and high Ag^+^ release coatings, respectively, corresponding to an approximately twofold increase in silver ion release for the high-release coating (Fig. 4D).

Furthermore, the concentrations of released metal ions within a 2-μL virus-containing droplet deposited on each coating surface were estimated. As a result, the estimated Cu^2+^ concentration within the droplet on the high Cu^2+^ release coating was approximately threefold higher than that on the low-release coating at the same time points. A similar trend was observed for Ag^+^, with the high-release coating yielding approximately twofold higher estimated droplet concentrations than the low-release coating (Fig. 4E).

### 3.4 Survival time–based performance evaluation of antiviral coating surfaces

**Fig. 5:**
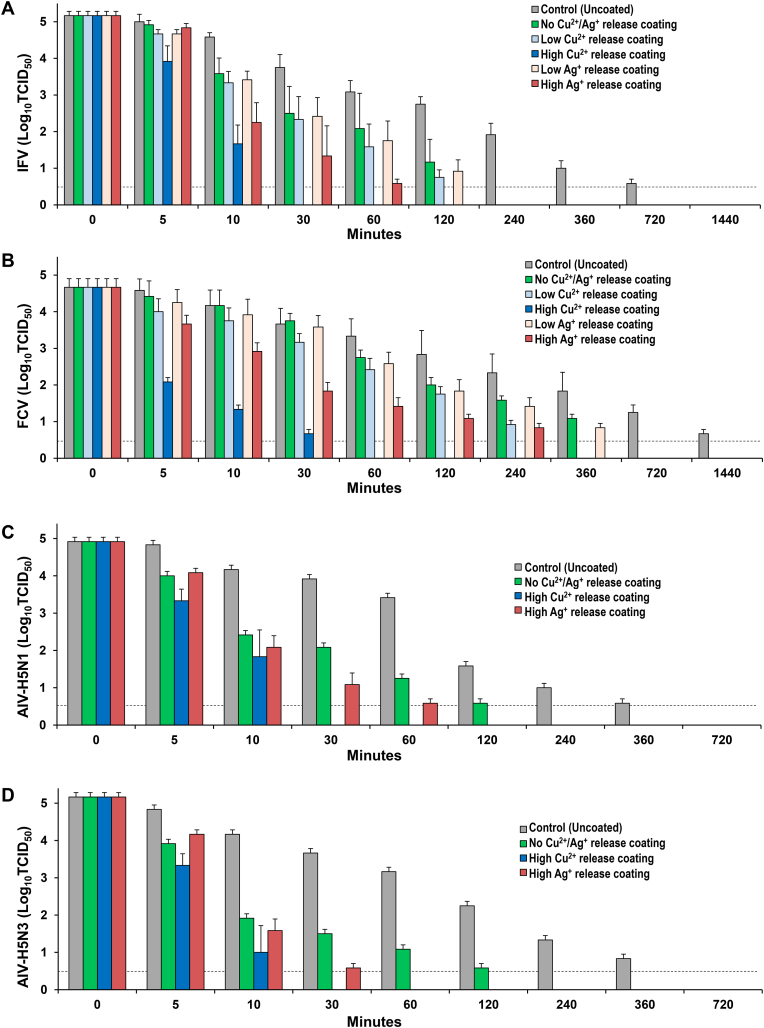
**(A, B) Time-course changes in the titers of (A) influenza virus (IFV) and (B) feline calicivirus (FCV) remaining on antiviral coating surfaces with different metal ion release profiles (no release, low Cu^2+^ release, high Cu^2+^ release, low Ag** + **release, and high Ag** + **release). (C, D) Time-course changes in the titers of (C) highly pathogenic avian influenza virus (AIV-H5N1) and (D) low pathogenic avian influenza virus (AIV-H5N3) remaining on selected antiviral coating surfaces (no release, high Cu^2+^ release, and high Ag** + **release).** For each measurement, three independent experiments were conducted, and the results are expressed as the mean ± standard error. Data points below the detection limit are omitted, and dotted horizontal lines indicate the detection limit titers.

On hydrophilic coating surfaces that did not release copper or silver ions (i.e., hydrophilic coating alone), the amounts of infectious IFV and FCV remaining on the surface were reduced compared with those on uncoated glass surfaces. This reduction was further enhanced on hydrophilic coating surfaces that released either copper or silver ions (Fig. 5A and B). Notably, all hydrophilic coating surfaces were adjusted to the same water contact angle of 30°.

Based on the data shown in Fig. 5A and B, viral survival time and survival time reduction rate were calculated (Table 1 and Supplementary Figs. S5 and S6). On hydrophilic coating alone, the survival times of IFV and FCV were significantly shorter than those on uncoated glass surfaces, with survival time reduction rates of 79.1% and 69.5%, respectively. The previously described reduction in surface virus density associated with increased surface hydrophilicity (i.e., lower contact angle) contributed to these decreases in viral survival time.

**Table 1:** Survival time of influenza virus (IFV) and feline calicivirus (FCV) on each antiviral coating surface.

	Estimated survival time (95% CI)		Survival time reduction rate	
	IFV	FCV	IFV	FCV
**Control (Uncoated)**	1100 min (850-1400 min)	2700 min (1800-4500 min)	-	-
**No Cu^2+^/Ag** + **release coating**	230 min (150-420 min)	820 min (610-1200 min)	79.1%	69.5%
**Low Cu^2+^ release coating**	180 min (130-270 min)	470 min (340-690 min)	83.3%	82.7%
**High Cu^2+^ release coating**	26 min (19-50 min)	48 min (37-68 min)	97.6%	98.2%
**Low Ag** + **release coating**	200 min (150-290 min)	710 min (520-1000 min)	81.6%	73.6%
**High Ag** + **release coating**	77 min (50-150 min)	300 min (230-410 min)	92.9%	89.0%

On low Cu^2+^ release coating and low Ag ^+^ release coating, further reductions in IFV and FCV survival times were observed compared with hydrophilic coating alone; however, these differences were not statistically significant. By contrast, on high Cu^2+^ release coating, the survival times of IFV and FCV were markedly shortened, with survival time reduction rates of 97.6% and 98.2%, respectively. These survival times were significantly shorter not only than those on hydrophilic coating alone but also than those on low Cu^2+^ release coating. Similarly, on high Ag^+^ release coating, the survival times of IFV and FCV were substantially reduced, with survival time reduction rates of 92.9% and 89.0%, respectively. These reductions were also statistically significant compared with both hydrophilic coating alone and low Ag^+^ release coating (Table 1).

Finally, direct comparison of high Cu^2+^ release coating and high Ag ^+^ release coating revealed that high Cu^2+^ release coating resulted in significantly shorter survival times and greater survival time reduction rates for both IFV and FCV. This finding indicates that, when combined with enhanced surface hydrophilicity, copper ion–releasing coatings provide superior antiviral performance compared with silver ion–releasing coatings, which is consistent with the earlier observation that copper ions exhibit significantly higher antiviral activity than silver ions.

### 3.5 Performance evaluation of antiviral coating surfaces against avian influenza viruses (AIVs)

As an additional analysis to confirm the general applicability of the evaluation system used in this study, the antiviral performance of coating surfaces was assessed against highly pathogenic avian influenza virus AIV-H5N1 and low pathogenic avian influenza virus AIV-H5N3. Consistent with the results obtained for IFV, hydrophilic coating alone resulted in reduced amounts of infectious AIV-H5N1 and AIV-H5N3 remaining on the surface compared with uncoated glass surfaces. This reduction was further enhanced on hydrophilic coatings that released either copper or silver ions (Fig. 5C and D).

On hydrophilic coating alone, the survival times of AIV-H5N1 and AIV-H5N3 were significantly shorter than those on uncoated glass surfaces, with survival time reduction rates of 69.7% and 79.3%, respectively. On high Cu^2+^ release coating, the survival times of AIV-H5N1 and AIV-H5N3 were substantially reduced, with survival time reduction rates of 94.9% and 96.6%, respectively. Similarly, on high Ag^+^ release coating, the survival times of AIV-H5N1 and AIV-H5N3 were also substantially reduced, with survival time reduction rates of 88.8% and 94.2%, respectively. These survival times were significantly shorter than those observed on hydrophilic coating alone (Table 2 and Supplementary Figs. S7 and S8).

**Table 2:** Survival time of avian influenza viruses (AIVs) on each antiviral coating surface.

	Estimated survival time (95% CI)		Survival time reduction rate	
	AIV-H5N1	AIV-H5N3	AIV-H5N1	AIV-H5N3
**Control (Uncoated)**	550 min (410-820 min)	710 min (540-990 min)	-	-
**No Cu^2+^/Ag** + **release coating**	170 min (120-270 min)	150 min (96-280 min)	69.7%	79.3%
**High Cu^2+^ release coating**	28 min (20-54 min)	24 min (16-59 min)	94.9%	96.6%
**High Ag** + **release coating**	62 min (43-110 min)	42 min (28-79 min)	88.8%	94.2%

AIV-H5N1, highly pathogenic avian influenza virus; AIV-H5N3, low pathogenic avian influenza virus.

Overall, these results were consistent with those obtained for IFV, indirectly supporting the robustness and general applicability of the present performance evaluation system.

## 4 Discussion

A central finding of this study is that viral persistence on surfaces can be quantitatively predicted by surface virus density under realistic drying conditions. This principle emerges from a dynamic evaluation framework that captures realistic microdroplet drying behavior, thereby complementing the standardized ISO 21702 methodology. ISO 21702 provides an important standardized approach for evaluating antiviral surface performance under controlled experimental conditions using a fixed 24 h endpoint (Fig. 1A) [[26], [27], [28], [29], [30], [31]]. By contrast, the evaluation framework established in this study was designed to quantify viral persistence dynamics during microdroplet drying and to investigate the interfacial factors governing viral survival under drying conditions (Fig. 1B). Moreover, this evaluation framework quantifies antiviral performance using dynamic parameters, namely viral survival time and survival time reduction rate. This approach enables robust comparison among antiviral coatings based on viral persistence dynamics, thereby providing a principle-based assessment of antiviral performance. Importantly, the survival time reduction rate enables antiviral performance to be expressed in an intuitive and practically meaningful manner. For example, a reduction in viral survival time from 20 h to 4 h corresponds to an 80% survival time reduction. Such expressions facilitate practical interpretation of antiviral performance across scientific, biomedical, and bioengineering contexts. This supports rational engineering and practical selection of antiviral coating technologies according to specific usage scenarios. For instance, in situations where the infection risk must be effectively eliminated by the following morning, an antiviral surface capable of reducing viral survival time to less than 6 h would be an appropriate choice.

Previous studies have reported that surface properties, such as water contact angle, may influence viral survival; however, the underlying mechanisms have remained unclear [[35], [36], [37], [38], [39]]. In this study, we validate the hypothesis that viral survival time is quantitatively controlled by surface virus density (Fig. 1C), as evidenced by the convergence of survival times across different inoculum volumes and droplet geometries when surface virus density was matched. This establishes surface virus density as a unifying and predictive interfacial design variable linking surface wettability to viral persistence on solid substrates. Several physicochemical and interfacial transport processes likely contribute to this behavior, including accelerated drying due to liquid film thinning and enhanced viral inactivation resulting from spatial redistribution of virus particles [[37], [38], [39]]. While these processes were not directly visualized in the present study, future studies employing surface analytical techniques such as X-ray Photoelectron Spectroscopy and zeta potential analysis may provide additional insight into surface compositional and electrostatic changes occurring at coating interfaces during droplet drying and evaporation. Although the individual contributions of these factors remain to be fully resolved, the present results demonstrate that surface virus density serves as a predictive and quantitative determinant of viral survival time under controlled drying conditions. Although additional physicochemical variables may modulate viral decay kinetics under specific environmental conditions, our results show that, under controlled drying conditions, surface virus density consistently predicts viral survival time across different inoculum volumes and droplet geometries, providing a quantitative basis for predictive antiviral surface design. Unlike previous studies that reported empirical correlations between wettability and antiviral performance, the present study identifies and experimentally validates surface virus density as the underlying quantitative variable that unifies these observations and enables predictive design of antiviral coatings.

Building on this principle, we established a wettability-engineered antiviral coating strategy that achieves substantial reductions in viral survival time solely through modulation of surface wettability, without the use of antiviral agents. This interface-based physical strategy offers two major advantages: first, because antiviral activity arises solely from surface properties, the antiviral function is expected to remain durable during prolonged use; second, the absence of released antiviral agents reduces potential concerns related to human toxicity or environmental accumulation. On hydrophilic coating surfaces without the release of antiviral agents such as copper or silver ions, survival time reduction rates of approximately 80% for IFV and 70% for FCV were achieved. Such reductions are expected to substantially reduce the persistence of infectious viruses on contaminated surfaces and thereby reduce contact transmission risk. Importantly, the antiviral performance of such agent-free, physically driven surfaces is difficult to evaluate using the conventional ISO 21702 methodology, which was primarily designed for endpoint-based assessment under persistently wet conditions. The evaluation framework established in this study overcomes this limitation, thereby enabling the development of antiviral surfaces based on physical strategies.

Many antiviral coatings combine a matrix material with antiviral agents; however, systematic understanding of the optimal agent type and its release kinetics remains limited. Here, antiviral coating surfaces were engineered with high- or low-ion-release profiles by altering the composition of vanadate glass particles incorporated into the coating matrix, thereby enabling quantitative correlation of copper and silver ion release behavior with antiviral performance as evaluated by viral survival time reduction. To approximate small-droplet contamination conditions on environmental surfaces, a 2 μL microdroplet deposited on the surface was modeled, and ion concentrations within the droplet were estimated 1–5 min after initial elution. High-release coatings produced approximately threefold higher copper ion concentrations and twofold higher silver ion concentrations than low-release coatings. Antiviral surface performance evaluations showed that survival times of IFV and FCV on high-release surfaces were significantly shorter than those on low-release surfaces. Because droplets deposited on surfaces typically dry within 5–10 min under the indoor drying conditions examined in this study, these results show that the amount of antiviral agent released during this short initial period plays a critical role in shortening viral survival time. Accordingly, within the formulation range examined in the present study, antiviral performance scales with the initial elution rate, demonstrating that early-stage release kinetics constitute a key engineering parameter governing antiviral efficacy under rapid-drying conditions. From a practical perspective, the safety and environmental impact of ion-releasing coatings should also be considered. The coating system investigated here was designed to achieve antiviral activity through controlled early-stage ion release at relatively low levels, thereby minimizing the amount of released material required for antiviral efficacy. Nevertheless, comprehensive evaluation of long-term cytotoxicity, environmental fate, and life-cycle impacts will be important for future implementation of such coating technologies.

For both enveloped (IFV) and non-enveloped (FCV) viruses, copper ion–releasing coatings exhibited stronger antiviral activity than silver ion–releasing coatings, consistent with previous reports [32,53]. This difference was directly reflected in antiviral coating performance, as copper ion high-release surfaces achieved greater survival time reduction rates than silver ion high-release surfaces. By contrast, when infection control targets extend beyond viruses to include bacteria and fungi, silver ion–based systems may offer advantages for multifunctional antimicrobial interface applications [54,55].

Ultimately, high antiviral performance is achieved through synergistic integration of wettability engineering (physical control) and rapid ion release (chemical control). As illustrated in Fig. 1D, hydrophilic coating interfaces were engineered by incorporating silica nanoparticles into the coating matrix. Subsequently, metal ion–releasing hydrophilic coatings were fabricated by incorporating V_2_O_5_-based glass particles capable of releasing copper or silver ions. Hydrophilic surfaces based solely on the physical regulation already exhibited substantial antiviral performance, achieving survival time reduction rates of approximately 70–80% even in the absence of antiviral agents. Hydrophilic surfaces capable of rapidly releasing copper ions further accelerated viral inactivation kinetics and shortened survival times, resulting in survival time reduction rates of 97.6% for IFV and 98.2% for FCV. These results demonstrate that physical redistribution and chemical inactivation function as complementary and coupled interface engineering mechanisms, enabling antiviral performance that exceeds that achievable by single-mechanism coating systems. Importantly, while antiviral coatings based on metal ion release have been previously reported, the objective of the present study was not merely to develop another antiviral coating formulation. Rather, this study establishes a quantitative framework that links interfacial properties to viral persistence and identifies surface virus density and early-stage ion release kinetics as predictive design variables for antiviral coating engineering.

The antiviral coating surfaces developed in this study rapidly inactivate surface-associated viruses and, unlike conventional short-duration disinfection approaches, are expected to provide sustained antiviral functionality over extended periods ranging from months to years. Consequently, such antiviral coatings can contribute to reducing contact transmission risks across healthcare, agricultural, and community settings. Notably, the demonstrated efficacy against both low- and highly pathogenic avian influenza viruses (AIV-H5N3 and AIV-H5N1), comparable to that observed for IFV, highlights the potential of these antiviral coatings as durable antiviral interface materials for emerging zoonotic threats [56,57].

Several limitations of this study should be acknowledged. First, glass was used as the model substrate material throughout this study. In practical applications, antiviral coatings may be applied to a wide variety of surfaces, including stainless steel, polymers, and other metals. However, once coated, key surface properties such as contact angle and antiviral agent release behavior are expected to be predominantly governed by the coating layer rather than the underlying substrate. Accordingly, glass substrates with an initial contact angle of approximately 60° were selected as representative model substrates in this study. Notably, preliminary evaluations conducted on polymer substrates yielded similar antiviral performance after coating. Because uncoated hydrophobic surfaces with higher contact angles generally exhibit longer viral survival times, such surfaces may exhibit even larger reductions in viral survival time following hydrophilic coating treatment. Because the proposed coating strategy is based on controllable coating properties rather than substrate-specific characteristics, it may be applicable to coating systems used on a wide range of practical materials, including polymers, stainless steel, painted surfaces, and other commonly encountered environmental substrates. Potential applications include healthcare facilities, livestock production environments, public transportation systems, and community settings. Nevertheless, systematic validation across diverse substrate materials remains an important subject for future investigation. In addition, although only representative enveloped and non-enveloped viruses were examined, the convergence of survival times under matched surface virus density suggests that surface virus density may represent a broadly applicable determinant of viral persistence beyond the specific viral models examined here.

Second, this study focused on antiviral coatings containing vanadate glass particles that release either copper or silver ions; however, the conceptual framework presented here may be transferable beyond the specific material composition and release mechanism investigated here. Other antiviral agents and alternative release mechanisms may also be incorporated within this framework, provided that the agents are released rapidly and reach concentrations sufficient to exert measurable antiviral activity (e.g., approximately a 1-log reduction). Accordingly, the conceptual framework established here may serve as a generalizable interface engineering strategy for diverse antiviral coating architectures beyond the specific system investigated. In addition, ion release behavior was evaluated using deionized water as a standardized model medium. Biological fluids and environmental organic matter may influence metal ion release through complex chemical interactions. Therefore, further studies are needed to determine how the relationships identified in the present study are affected under biologically relevant conditions.

Third, viral survival time and survival time reduction rate were employed as dynamic metrics for evaluating antiviral surface performance. Viral survival time was estimated by regression modeling of viral titer decay curves, as shown in Supplementary Fig. S1–S8. In the present study, measurements were continued until viral infectivity fell below the detection limit under all tested conditions, thereby minimizing uncertainties associated with extrapolation near the detection threshold. Alternatively, viral half-life and half-life reduction rate could also be used as performance metrics. The same regression analysis can also be used to estimate viral half-lives. Because both parameters are derived from identical datasets and analytical procedures, the overall conclusions regarding antiviral performance remain unchanged regardless of which parameter is used. In this study, viral survival time was selected because it offers a more intuitive representation of antiviral performance under realistic drying conditions; however, either metric can therefore be used within the proposed evaluation framework without altering the principal conclusions.

Fourth, although the relationship between surface wettability, surface virus density, and viral survival time was consistently observed throughout this study, the underlying physicochemical processes responsible for this relationship, including virus redistribution and drying dynamics, were not directly visualized. Therefore, the proposed mechanism is supported by indirect experimental evidence rather than direct observation. Future studies combining imaging-based approaches with surface analytical techniques, such as X-ray Photoelectron Spectroscopy and zeta potential analysis, may provide further mechanistic insight into how these interfacial processes contribute to viral persistence.

In conclusion, we establish a quantitative evaluation framework for antiviral surfaces, coating systems, and functional interfaces that captures realistic microdroplet drying dynamics and defines antiviral performance using viral survival time and survival time reduction rate. Using this framework, we show that viral survival time is strongly governed by surface virus density and establish surface virus density as a unifying physical determinant that enables predictive control of viral persistence through surface wettability. We further show that early-stage release kinetics of antiviral agents, as quantified by the initial elution rate, constitute a key chemical determinant of antiviral efficacy under rapid-drying conditions. By integrating interfacial wettability engineering with rapid antiviral agent release, we demonstrate synergistic control of viral persistence via coupled physical and chemical design axes. The optimized coating system achieves survival time reductions of up to 98% under the tested conditions. Collectively, these findings provide a quantitative evaluation methodology and generalizable design principles for antiviral surfaces, thereby establishing a foundation for the rational engineering of next-generation antiviral coatings for mitigation of contact transmission risks in healthcare and community environments.

## CRediT authorship contribution statement

**Ryohei Hirose:** Conceptualization, Data curation, Formal analysis, Funding acquisition, Investigation, Methodology, Project administration, Resources, Software, Supervision, Validation, Visualization, Writing – original draft, Writing – review & editing. **Saori Morita:** Data curation, Formal analysis, Funding acquisition, Methodology, Resources. **Shizuka Kanawa:** Data curation, Formal analysis, Resources. **Akinobu Sai:** Data curation, Formal analysis. **Taku Kano:** Data curation, Formal analysis. **Takumi Minamiyama:** Data curation, Formal analysis. **Satomi Isono:** Data curation, Formal analysis. **Takaaki Nakaya:** Supervision, Validation.

## Declaration of competing interest

The authors declare the following financial interests/personal relationships which may be considered as potential competing interests: Ryohei Hirose reports financial support was provided by Takahashi Industrial and Economic Research Foundation. Ryohei Hirose reports financial support was provided by Takeda Science Foundation. Ryohei Hirose reports financial support was provided by Kaketsuken Kikuchi Laboratory. Ryohei Hirose reports financial support was provided by Food Science Institute Foundation. Ryohei Hirose reports financial support was provided by The Toyo Suisan Foundation. Ryohei Hirose reports a relationship with HardoLass Holdings Company that includes: funding grants. Dr. Hirose received a collaboration research fund from HardoLass Holdings Company, Limited. The funding agency has no influence on the study design or has no conflict of interest. All other authors disclosed no financial relationships relevant to this publication. If there are other authors, they declare that they have no known competing financial interests or personal relationships that could have appeared to influence the work reported in this paper.

The logarithm of elapsed time was used as the explanatory variable (X-axis), and the logarithm of the viral titer of IFV or FCV was used as the response variable (Y-axis). Least-squares linear regression analysis was performed for each virus to generate a curve of regression (see also Supplementary Figs. S5–S6). The measurement limits of the titers of IFV and FCV were 10°^.5^ TCID_50_; therefore, the survival times of IFV and FCV were defined as the X values when the Y values of the regression curves were 0.5.

$$Survival\;time\;reduction\;rate=100\times \frac{Survival\;time\;on\;control\;surface-survival\;time\;on\;coated\;surface}{Survival\;time\;on\;control\;surface}$$

See Supplementary Table S1 for log reduction values at representative time points on each surface.

The logarithm of elapsed time was used as the explanatory variable (X-axis), and the logarithm of the viral titer was used as the response variable (Y-axis). Least-squares linear regression analysis was performed for each virus to generate a curve of regression (see also Supplementary Figs. S7–S8). The measurement limits of the titers of AIV-H5N1 and AIV-H5N3 were 10°^.5^ TCID_50_; therefore, the survival times of AIV-H5N1 and AIV-H5N3 were defined as the X values when the Y values of the regression curves were 0.5.

AIV-H5N1, highly pathogenic avian influenza virus; AIV-H5N3, low pathogenic avian influenza virus.

$$Survival\;time\;reduction\;rate=100\times \frac{Survival\;time\;on\;control\;surface-survival\;time\;on\;coated\;surface}{Survival\;time\;on\;control\;surface}$$

See Supplementary Table S2 for log reduction values at representative time points on each surface.

## Data Availability

Data will be made available on request.
