# Supplementary material for: Synergistic control of viral persistence via wettability and ion release on antiviral coatings
Source: Mater Today Bio. 2026 Jul 13;39:103449. doi: 10.1016/j.mtbio.2026.103449 (PMC13400412; doi:10.1016/j.mtbio.2026.103449)
Supplement: Multimedia component 1 [file mmc1.pdf]

Supplementary material

Synergistic Control of Viral Persistence via Wettability and Ion Release on Antiviral Coatings

Ryohei Hirose, Saori Morita, Shizuka Kanawa, Akinobu Sai, Taku Kano, Takumi Minamiyama, Satomi Isono, Takaaki Nakaya

Correspondence to: ryo-hiro@koto.kpu-m.ac.jp (Ryohei Hirose)

Table of Contents

1. **Supplementary Figure S1.**
2. **Supplementary Figure S2.**
3. **Supplementary Figure S3.**
4. **Supplementary Figure S4.**
5. **Supplementary Figure S5.**
6. **Supplementary Figure S6.**
7. **Supplementary Figure S7.**
8. **Supplementary Figure S8.**
9. **Supplementary Table S1.**
10. **Supplementary Table S2.**

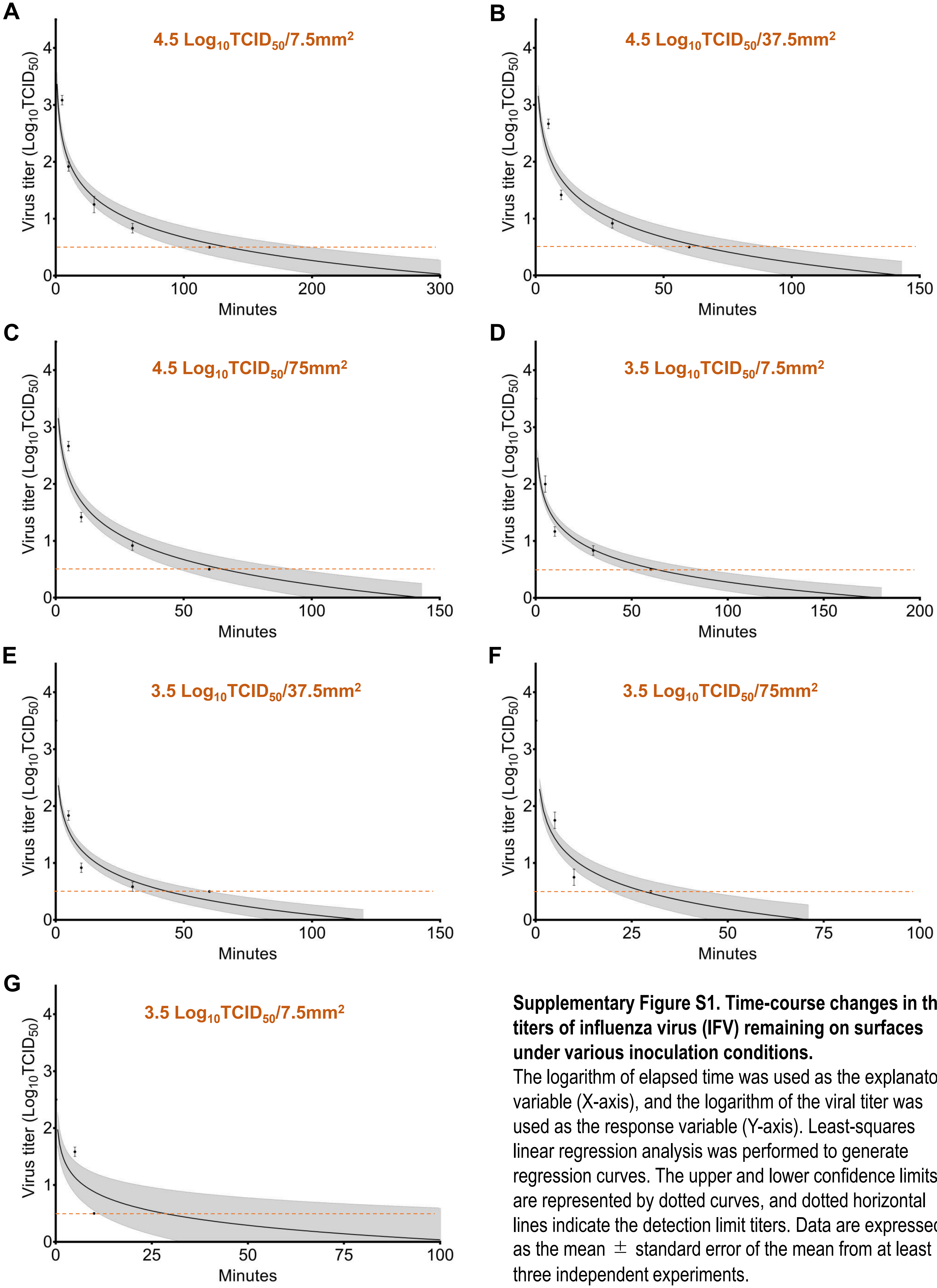

Supplementary Figure S1. Time-course changes in the titers of influenza virus (IFV) remaining on surfaces under various inoculation conditions.
The logarithm of elapsed time was used as the explanatory variable (X-axis), and the logarithm of the viral titer was used as the response variable (Y-axis). Least-squares linear regression analysis was performed to generate regression curves. The upper and lower confidence limits are represented by dotted curves, and dotted horizontal lines indicate the detection limit titers. Data are expressed as the mean ± standard error of the mean from at least three independent experiments.

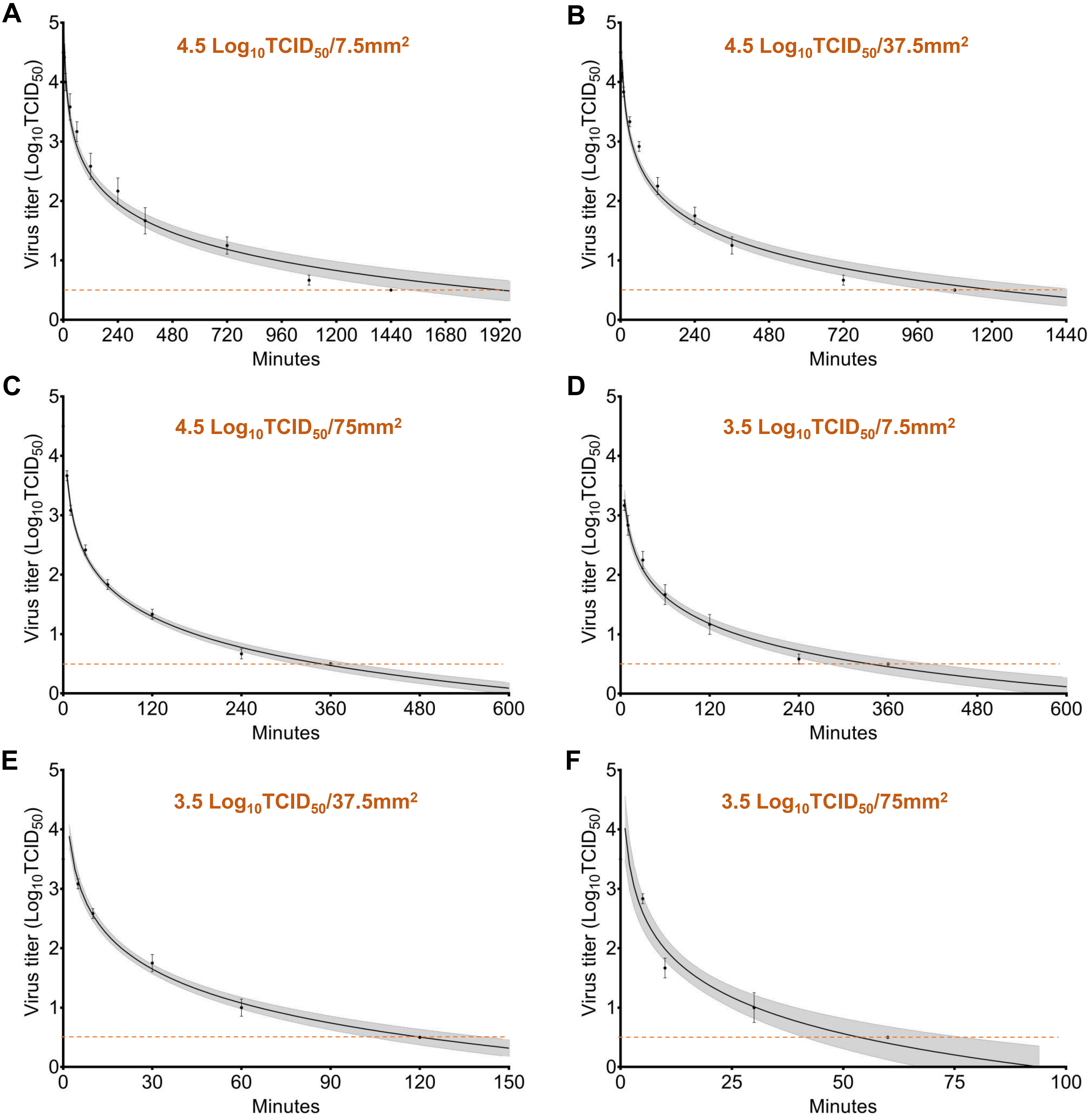

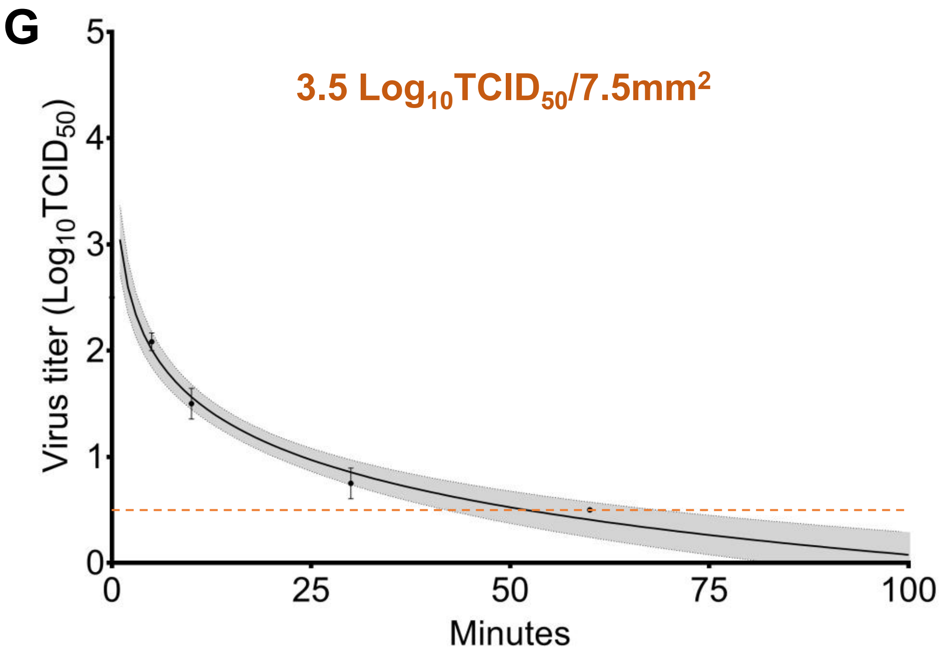

Supplementary Figure S2. Time-course changes in the titers of feline calicivirus (FCV) remaining on surfaces under various inoculation conditions.
The logarithm of elapsed time was used as the explanatory variable (X-axis), and the logarithm of the viral titer was used as the response variable (Y-axis). Least-squares linear regression analysis was performed to generate regression curves. The upper and lower confidence limits are represented by dotted curves, and dotted horizontal lines indicate the detection limit titers. Data are expressed as the mean ± standard error of the mean from at least three independent experiments.

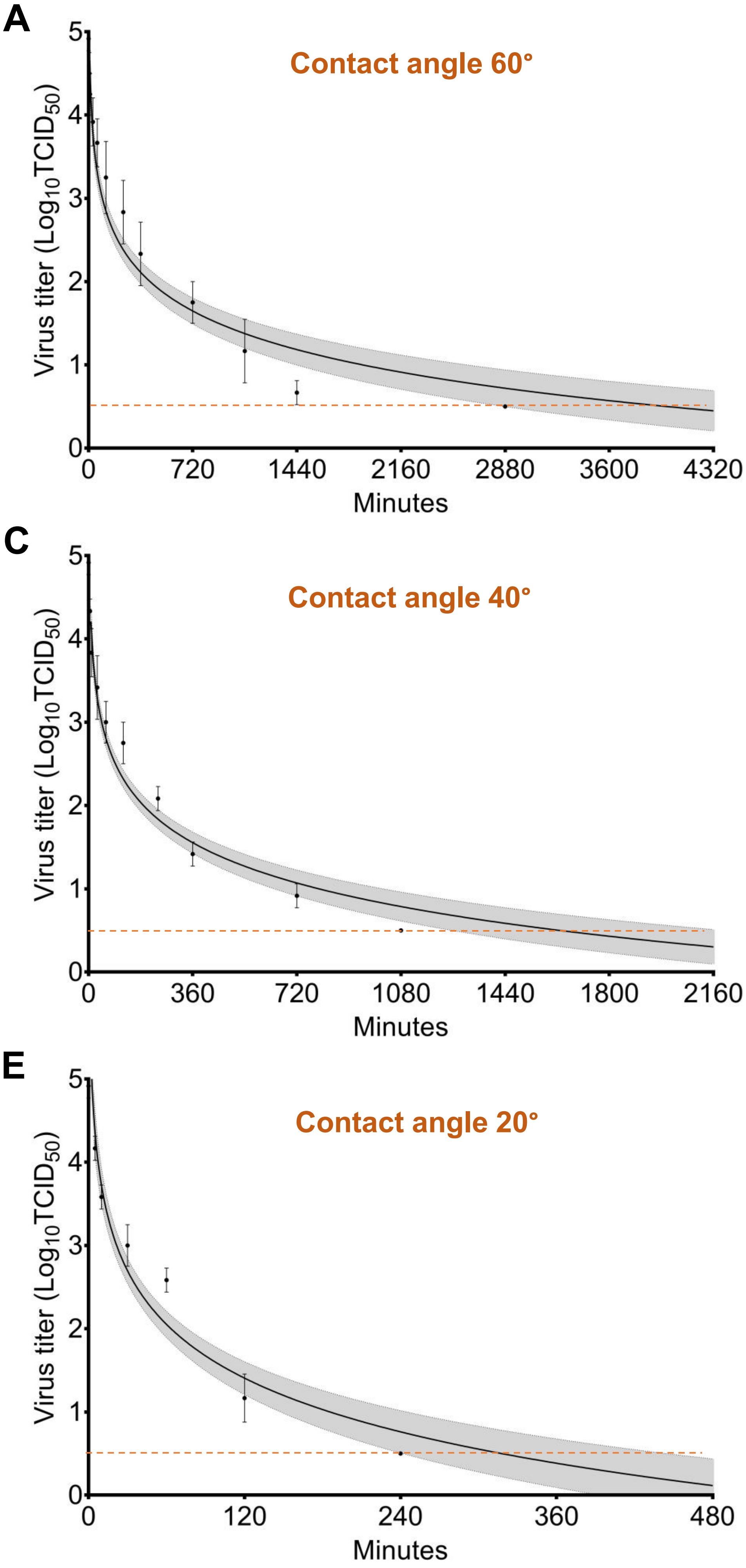

Supplementary Figure S3. Time-course changes in the titers of influenza virus (IFV) remaining on coated surfaces with contact angles adjusted to 20, 30, 40, 50, and 60° .

The logarithm of elapsed time was used as the explanatory variable (X-axis), and the logarithm of the viral titer was used as the response variable (Y-axis). Least-squares linear regression analysis was performed to generate regression curves. The upper and lower confidence limits are represented by dotted curves, and dotted horizontal lines indicate the detection limit titers. Data are expressed as the mean ± standard error of the mean from at least three independent experiments.

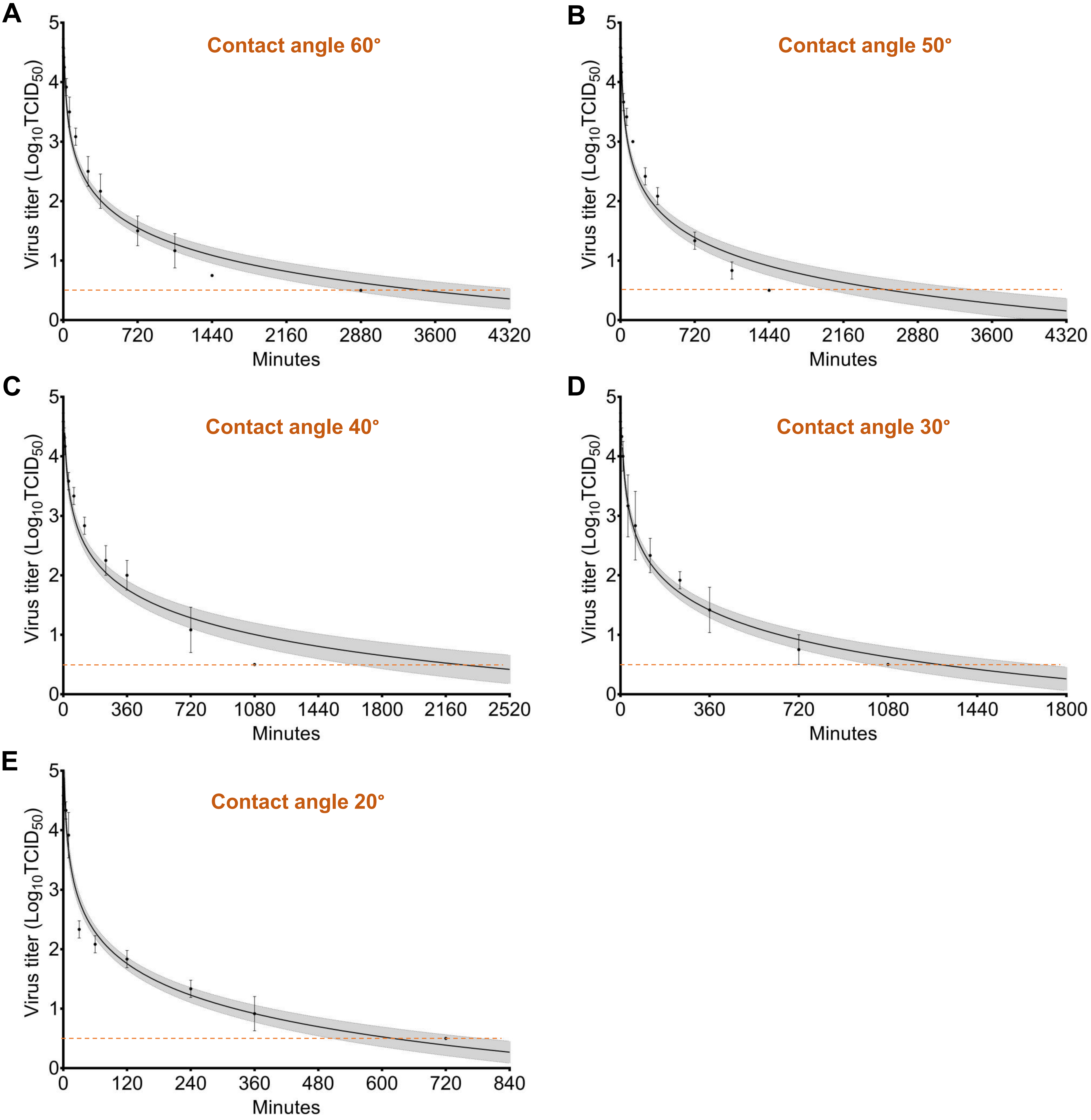

Supplementary Figure S4. Time-course changes in the titers of feline calicivirus (FCV) remaining on coated surfaces with contact angles adjusted to 20, 30, 40, 50, and 60° .
The logarithm of elapsed time was used as the explanatory variable (X-axis), and the logarithm of the viral titer was used as the response variable (Y-axis). Least-squares linear regression analysis was performed to generate regression curves. The upper and lower confidence limits are represented by dotted curves, and dotted horizontal lines indicate the detection limit titers. Data are expressed as the mean ± standard error of the mean from at least three independent experiments.

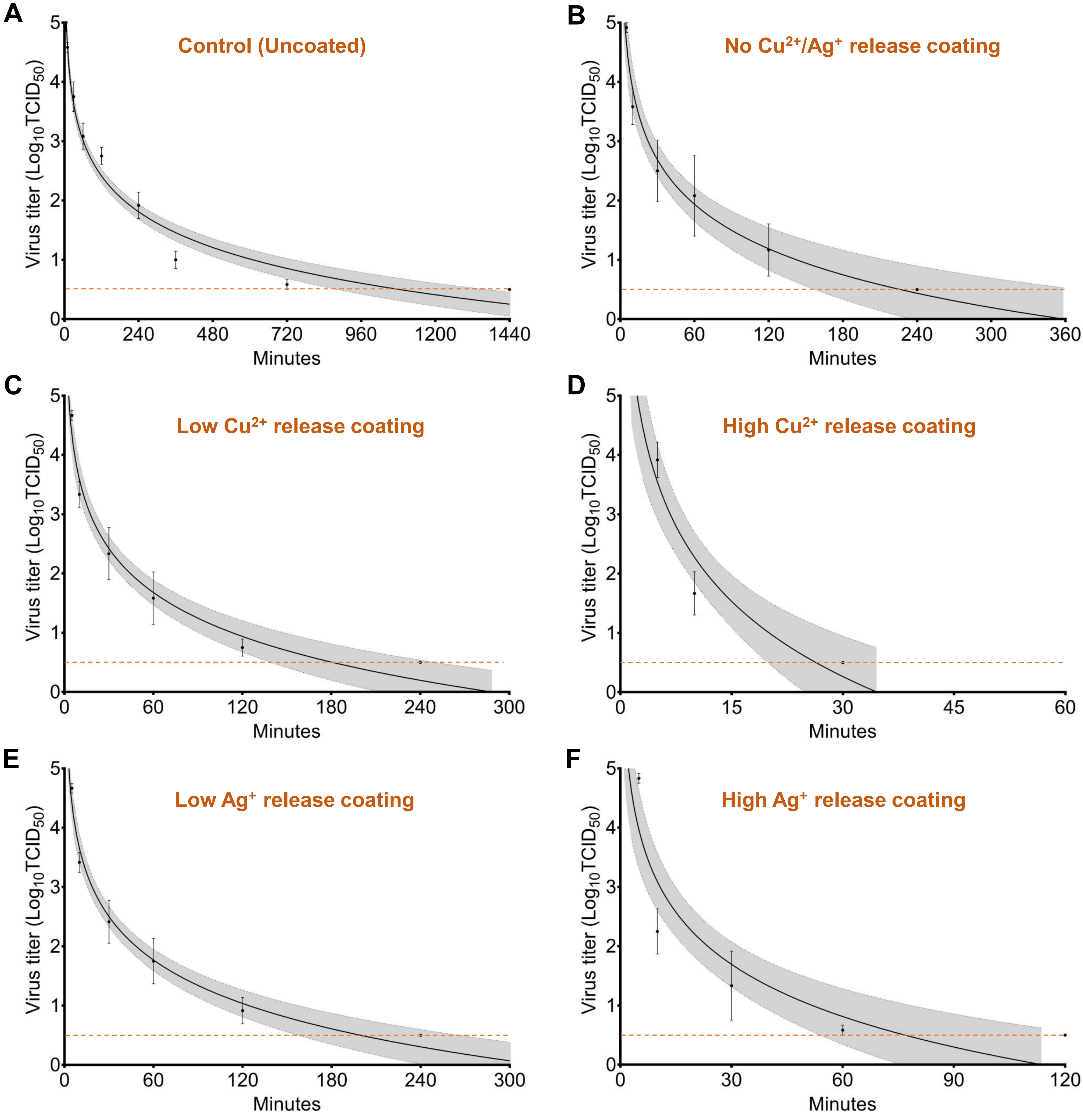

Supplementary Figure S5. Time-course changes in the titers of influenza virus (IFV) remaining on antiviral coating surfaces with different metal ion release properties (no release, low Cu^{2+} release, high Cu^{2+} release, low Ag^{+} release, and high Ag^{+} release).
The logarithm of elapsed time was used as the explanatory variable (X-axis), and the logarithm of the viral titer was used as the response variable (Y-axis). Least-squares linear regression analysis was performed to generate regression curves. The upper and lower confidence limits are represented by dotted curves, and dotted horizontal lines indicate the detection limit titers. Data are expressed as the mean ± standard error of the mean from at least three independent experiments.

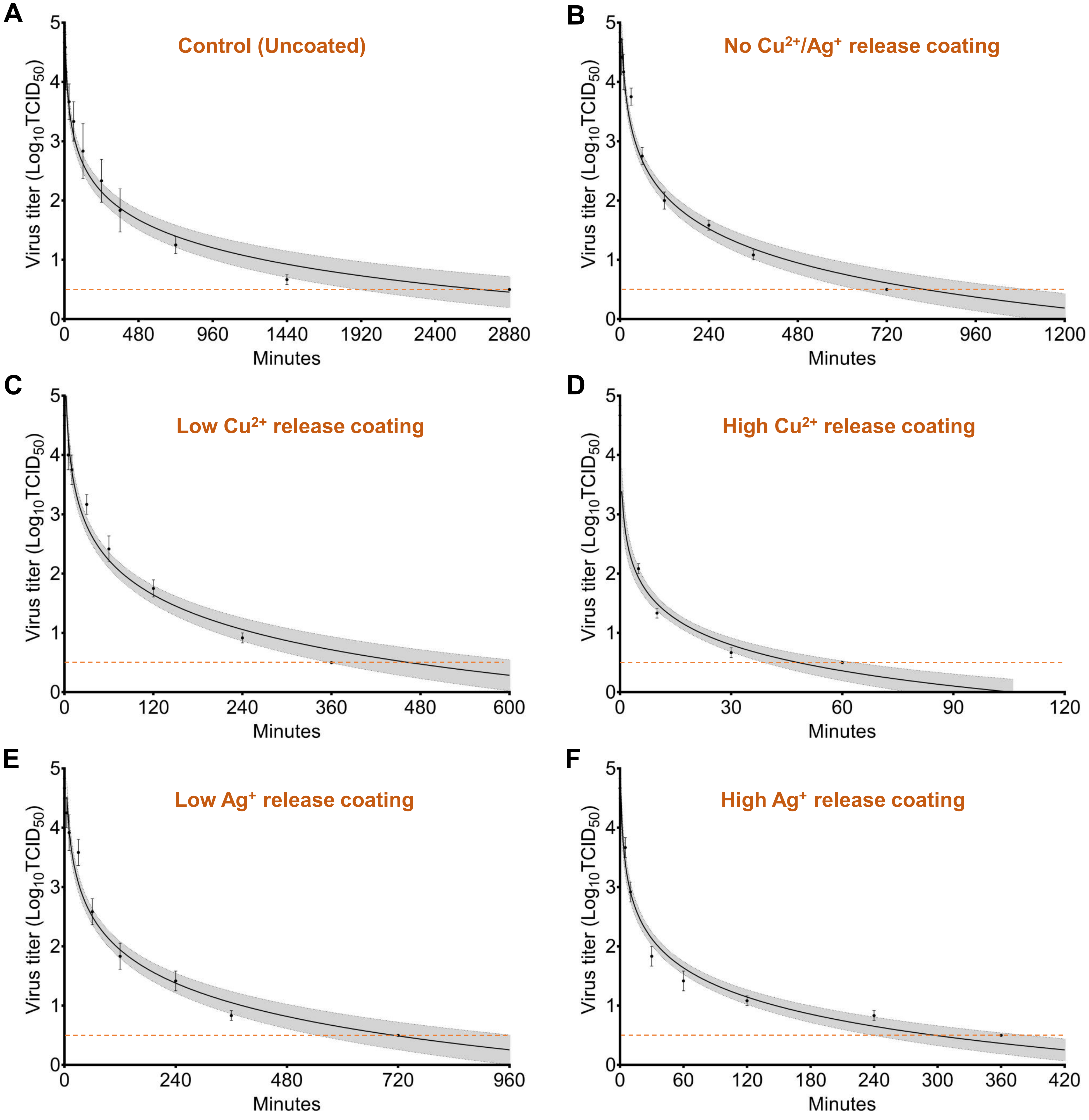

Supplementary Figure S6. Time-course changes in the titers of feline calicivirus (FCV) remaining on antiviral coating surfaces with different metal ion release properties (no release, low Cu^{2+} release, high Cu^{2+} release, low Ag^{+} release, and high Ag^{+} release).

The logarithm of elapsed time was used as the explanatory variable (X-axis), and the logarithm of the viral titer was used as the response variable (Y-axis). Least-squares linear regression analysis was performed to generate regression curves. The upper and lower confidence limits are represented by dotted curves, and dotted horizontal lines indicate the detection limit titers. Data are expressed as the mean $\pm$ standard error of the mean from at least three independent experiments.

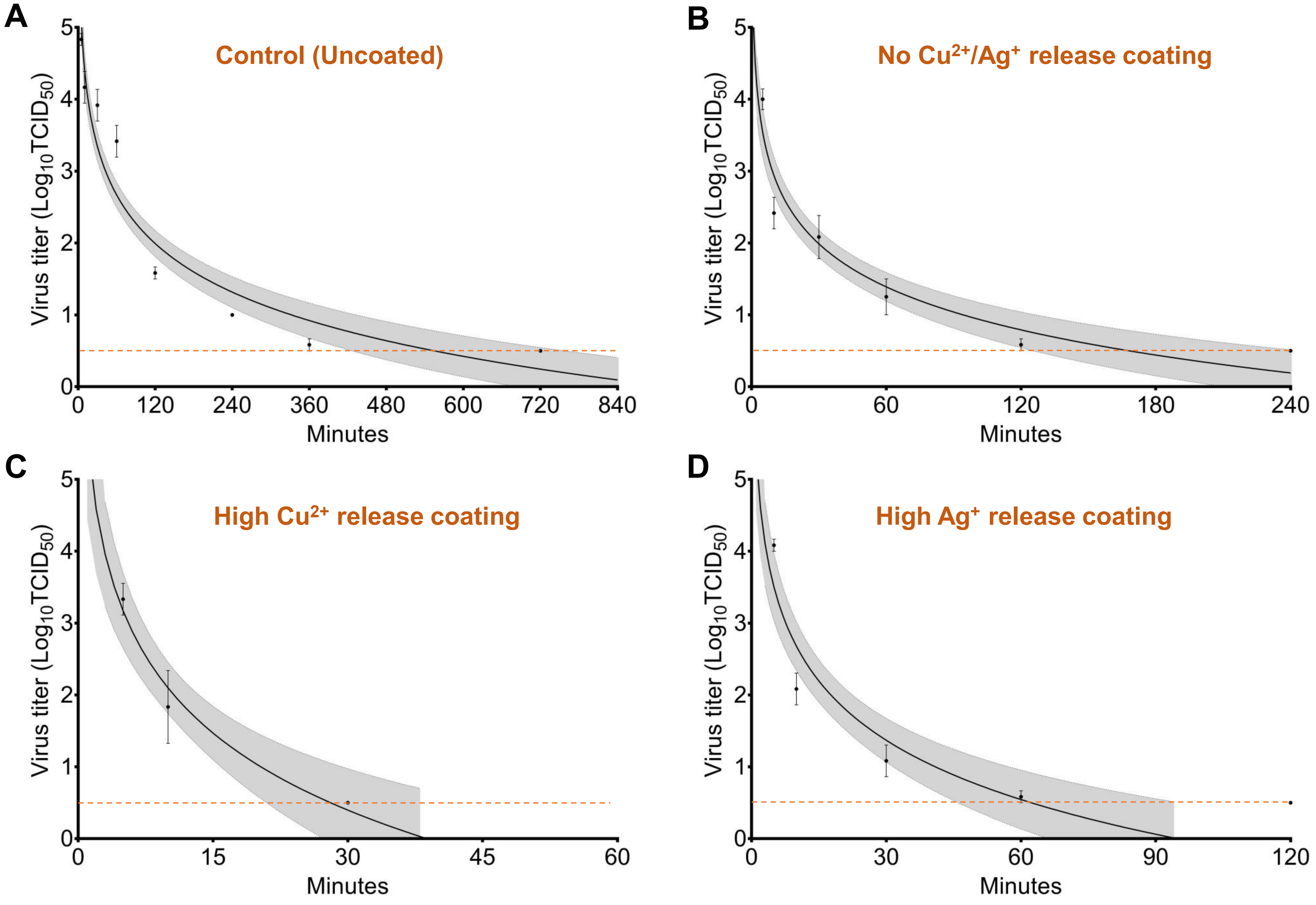

Supplementary Figure S7. Time-course changes in the titers of highly pathogenic avian influenza virus (AIV-H5N1) remaining on selected antiviral coating surfaces (no release, high Cu^{2+} release, and high Ag^{+} release).

The logarithm of elapsed time was used as the explanatory variable (X-axis), and the logarithm of the viral titer was used as the response variable (Y-axis). Least-squares linear regression analysis was performed to generate regression curves. The upper and lower confidence limits are represented by dotted curves, and dotted horizontal lines indicate the detection limit titers. Data are expressed as the mean ± standard error of the mean from at least three independent experiments.

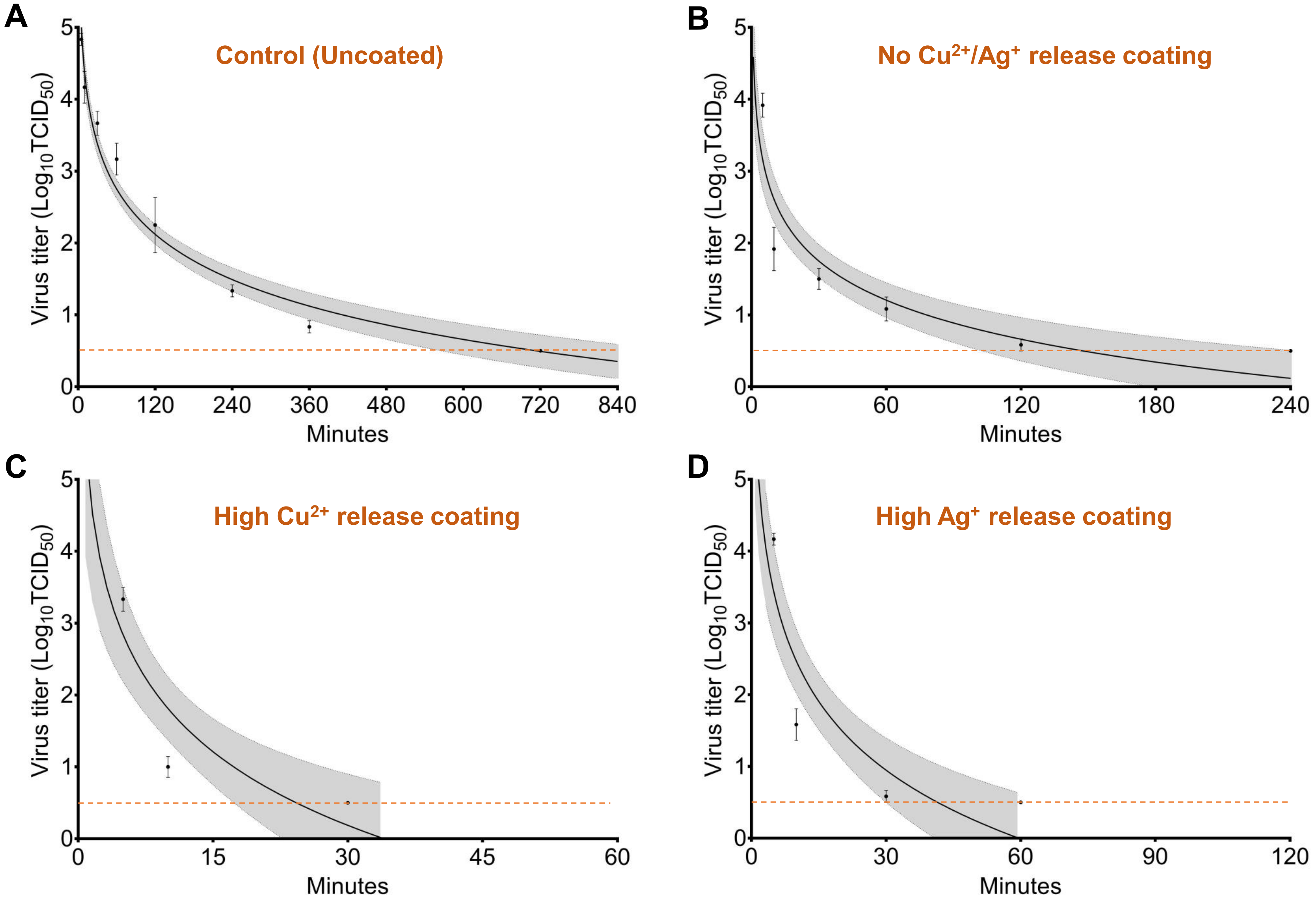

Supplementary Figure S8. Time-course changes in the titers of low pathogenic avian influenza virus (AIV-H5N3) remaining on selected antiviral coating surfaces (no release, high Cu^{2+} release, and high Ag^{+} release).
The logarithm of elapsed time was used as the explanatory variable (X-axis), and the logarithm of the viral titer was used as the response variable (Y-axis). Least-squares linear regression analysis was performed to generate regression curves. The upper and lower confidence limits are represented by dotted curves, and dotted horizontal lines indicate the detection limit titers. Data are expressed as the mean ± standard error of the mean from at least three independent experiments.

Supplementary Table S1. Log reduction values of influenza virus (IFV) and feline calicivirus (FCV) observed on each surface after 10 and 60 min of incubation.

	IFV		FCV	
	10 min	60 min	10 min	60 min
No Cu^{2+} and Ag^{+} release coating	1.6 ± 0.3	3.1 ± 0.7	0.5 ± 0.1	1.9 ± 0.1
Low Cu^{2+} release coating	1.9 ± 0.2	3.7 ± 0.4	0.8 ± 0.3	2.1 ± 0.2
High Cu^{2+} release coating	3.6 ± 0.4	> 4.5	3.3 ± 0.1	4.2 ± 0.2
Low Ag^{+} release coating	1.8 ± 0.2	3.5 ± 0.4	0.6 ± 0.3	1.9 ± 0.2
High Ag^{+} release coating	3 ± 0.4	> 4.5	1.8 ± 0.0	3.3 ± 0.1

The upper limit of detection for log reduction was 4.5, and values exceeding this limit were expressed as “> 4.5”.

Supplementary Table S2. Log reduction values of avian influenza viruses (AIVs) observed on each surface after 10 and 60 min of incubation.

	AIV-H5N1		AIV-H5N3	
	10 min	60 min	10 min	60 min
No Cu^{2+} and Ag^{+} release coating	2.6 ± 0.2	3.8 ± 0.3	3.1 ± 0.3	3.9 ± 0.2
High Cu^{2+} release coating	3.2 ± 0.5	> 4.5	4.0 ± 0.1	> 4.5
High Ag^{+} release coating	2.9 ± 0.2	4.4 ± 0.1	3.4 ± 0.2	> 4.5

The upper limit of detection for log reduction was 4.5, and values exceeding this limit were expressed as "> 4.5".
